# Supplementary material for: Insurer green finance under regulatory cap-and-trade mechanism associated with green/polluting production during a war
Source: PLoS One. 2023 Mar 16;18(3):e0282901. doi: 10.1371/journal.pone.0282901 (PMC10019747; doi:10.1371/journal.pone.0282901)
Supplement: S2 File — (DOCX) [file pone.0282901.s002.docx]

| Table 2 | | | | | | | |
| --- | --- | --- | --- | --- | --- | --- | --- |
| Responsiveness of the optimal guaranteed rate to $\theta$^*^ | | | | | | | |
|  | $(R(\%), L)$ | | | | | | |
| $\theta$ | (1.00, 307) | (1.20, 323) | (1.40, 334) | (1.60, 341) | (1.80, 345) | (2.00, 347) | (2.20, 348) |
|  | $S$ | | | | | | |
| 0.35 | 33.2286116082 | 34.8656187938 | 35.9315803983 | 36.5524137604 | 36.8455098949 | 36.9228049437 | 36.8926778750 |
| 0.40 | 33.2159685509 | 34.8522233655 | 35.9176578957 | 36.5381469684 | 36.8310371494 | 36.9082189959 | 36.8780252251 |
| 0.45 | 33.2033243913 | 34.8388267329 | 35.9037341197 | 36.5238788591 | 36.8165630609 | 36.8936316913 | 36.8633712105 |
| 0.50 | 33.1906791287 | 34.8254288952 | 35.8898090699 | 36.5096094322 | 36.8020876291 | 36.8790430295 | 36.8487158308 |
| 0.55 | 33.1780327626 | 34.8120298521 | 35.8758827456 | 36.4953386871 | 36.7876108536 | 36.8644530100 | 36.8340590855 |
| 0.60 | 33.1653852924 | 34.7986296029 | 35.8619551463 | 36.4810666233 | 36.7731327338 | 36.8498616325 | 36.8194009743 |
| 0.65 | 33.1527367174 | 34.7852281471 | 35.8480262716 | 36.4667932404 | 36.7586532693 | 36.8352688965 | 36.8047414967 |
|  | $\partial^{2}S/\partial R^{2}$ | | | | | | |
| 0.35 | - | -14.2116729594 | -11.0723653735 | -8.1498495252 | -5.3653730449 | -2.6705954448 | - |
| 0.40 | - | -14.2168734527 | -11.0781104407 | -8.1548452776 | -5.3689548582 | -2.6724431087 | - |
| 0.45 | - | -14.2219255432 | -11.0837346458 | -8.1597498527 | -5.3724757832 | -2.6742602317 | - |
| 0.50 | - | -14.2268298631 | -11.0892384301 | -8.1645635501 | -5.3759360076 | -2.6760469051 | - |
| 0.55 | - | -14.2315870474 | -11.0946222378 | -8.1692866716 | -5.3793357204 | -2.6778032215 | - |
| 0.60 | - | -14.2361977329 | -11.0998865151 | -8.1739195214 | -5.3826751122 | -2.6795292739 | - |
| 0.65 | - | -14.2406625588 | -11.1050317110 | -8.1784624055 | -5.3859543753 | -2.6812251563 | - |
|  | $\partial^{2}S/\partial R\partial\theta$ | | | | | | |
| 0.35→0.40 | 0.1039137432 | 0.0831117700 | 0.0601315011 | 0.0401484914 | 0.0258212383 | 0.0184305826 | - |
| 0.40→0.45 | 0.1019795760 | 0.0817712143 | 0.0592743941 | 0.0396560938 | 0.0255723935 | 0.0183039018 | - |
| 0.45→0.50 | 0.1000520446 | 0.0804347647 | 0.0584196273 | 0.0391648378 | 0.0253239403 | 0.0181772464 | - |
| 0.50→0.55 | 0.0981311934 | 0.0791024564 | 0.0575672260 | 0.0386747398 | 0.0250758887 | 0.0180506232 | - |
| 0.55→0.60 | 0.0962170661 | 0.0777743242 | 0.0567172149 | 0.0381858159 | 0.0248282486 | 0.0179240389 | - |
| 0.60→0.65 | 0.0943097058 | 0.0764504023 | 0.0558696186 | 0.0376980821 | 0.0245810298 | 0.0177975001 | - |
|  | $\partial R/\partial\theta$ | | | | | | |
| 0.35→0.40 | - | 0.0073118586 | 0.0075062344 | 0.0073782345 | 0.0074828891 | 0.0096687195 | - |
| 0.40→0.45 | - | 0.0071731367 | 0.0073813323 | 0.0072686105 | 0.0073861850 | 0.0095689197 | - |
| 0.45→0.50 | - | 0.0070350561 | 0.0072570092 | 0.0071594875 | 0.0072899049 | 0.0094695123 | - |
| 0.50→0.55 | - | 0.0068976149 | 0.0071332632 | 0.0070508638 | 0.0071940476 | 0.0093704967 | - |
| 0.55→0.60 | - | 0.0067608107 | 0.0070100921 | 0.0069427377 | 0.0070986118 | 0.0092718720 | - |
| 0.60→0.65 | - | 0.0066246415 | 0.0068874941 | 0.0068351075 | 0.0070035960 | 0.0091736373 | - |
| ^*^ Unless otherwise indicated, $\alpha=$ 0.9, $\beta=$ 0.25, $\theta=$ 0.50, $K_{h}=$ 78.13, $K_{l}=$ 14.66, $\alpha_{a}=$ 0.9, $\kappa=$ 0.75, $R_{B}=$ 2.41%, $R_{g}=$ 4.60%, $R_{c}=$ 5.10%, $R_{h}=$ 5.60%, $R_{l}=$ 5.10%, $\sigma_{h}=$ 0.2904, $\sigma_{l}=$ 0.2404, $b_{h}=$ 0.03, $b_{l}=$ 0.03, $\delta=$ 0.85, $c_{h}=$ 0.038, $c_{cap}=$ 0.022, $c_{l}=$ 0.60%, $R_{ci}=$ 0.5%, $R_{a}=$ 5.6%, and $\sigma_{a}=$ 29.04%. The shaded areas illustrate the optimal values and the comparative statics evaluated at the optimal guaranteed rate. | | | | | | | |

| Table 3 | | | | | | | |
| --- | --- | --- | --- | --- | --- | --- | --- |
| Responsiveness of $PUT_{lia}$ to $\theta$^*^ | | | | | | | |
|  | $(R(\%), L)$ | | | | | | |
| $\theta$ | (1.00, 307) | (1.20, 323) | (1.40, 334) | (1.60, 341) | (1.80, 345) | (2.00, 347) | (2.20, 348) |
|  | $PUT_{lia}$ | | | | | | |
| 0.35 | 373.1831311640 | 390.9814524294 | 403.2338123980 | 411.0715030050 | 415.6065056337 | 417.9421027098 | 419.1786395694 |
| 0.40 | 373.2837893871 | 391.0927559215 | 403.3524242544 | 411.1947330365 | 415.7323295784 | 418.0691694803 | 419.3062733672 |
| 0.45 | 373.3839794921 | 391.2035627241 | 403.4705201980 | 411.3174350958 | 415.8576187329 | 418.1956980907 | 419.4333673458 |
| 0.50 | 373.4837018019 | 391.3138731087 | 403.5881004639 | 411.4396093961 | 415.9823732990 | 418.3216887388 | 419.5599217026 |
| 0.55 | 373.5829566456 | 391.4236873530 | 403.7051652945 | 411.5612561578 | 416.1065934859 | 418.4471416299 | 419.6859366424 |
| 0.60 | 373.6817443586 | 391.5330057415 | 403.8217149389 | 411.6823756085 | 416.2302795100 | 418.5720569764 | 419.8114123774 |
| 0.65 | 373.7800652819 | 391.6418285653 | 403.9377496533 | 411.8029679828 | 416.3534315951 | 418.6964349981 | 419.9363491271 |
|  | $\partial PUT_{lia}/\partial\theta:$direct effect | | | | | | |
| 0.35→0.40 | 2.0131644634 | 2.2260698407 | 2.3722371289 | 2.4646006292 | 2.5164788940 | 2.5413354102 | 2.5526759568 |
| 0.40→0.45 | 2.0038020987 | 2.2161360535 | 2.3619188712 | 2.4540411861 | 2.5057830892 | 2.5305722082 | 2.5418795713 |
| 0.45→0.50 | 1.9944461956 | 2.2062076910 | 2.3516053188 | 2.4434860064 | 2.4950913219 | 2.5198129632 | 2.5310871358 |
| 0.50→0.55 | 1.9850968754 | 2.1962848858 | 2.3412966113 | 2.4329352342 | 2.4844037381 | 2.5090578217 | 2.5202987968 |
| 0.55→0.60 | 1.9757542588 | 2.1863677701 | 2.3309928882 | 2.4223890129 | 2.4737204831 | 2.4983069298 | 2.5095147004 |
| 0.60→0.65 | 1.9664184663 | 2.1764564756 | 2.3206942885 | 2.4118474857 | 2.4630417023 | 2.4875604335 | 2.4987349924 |
|  | $(\partial PUT_{lia}/\partial R)(\partial R/\partial\theta):$indirect effect | | | | | | |
| 0.35→0.40 | - | 0.6506940421 | 0.4598454302 | 0.2891415968 | 0.1696746084 | 0.1129111654 | - |
| 0.40→0.45 | - | 0.6387307529 | 0.4524634288 | 0.2850134400 | 0.1675776372 | 0.1118051669 | - |
| 0.45→0.50 | - | 0.6268088423 | 0.4451071185 | 0.2808994466 | 0.1654875357 | 0.1107023568 | - |
| 0.50→0.55 | - | 0.6149282750 | 0.4377764714 | 0.2767996023 | 0.1634042997 | 0.1096027380 | - |
| 0.55→0.60 | - | 0.6030890161 | 0.4304714601 | 0.2727138926 | 0.1613279255 | 0.1085063135 | - |
| 0.60→0.65 | - | 0.5912910319 | 0.4231920575 | 0.2686423032 | 0.1592584089 | 0.1074130860 | - |
|  | $dPUT_{lia}/d\theta:$total effect | | | | | | |
| 0.35→0.40 | - | 2.8767638828 | 2.8320825592 | 2.7537422260 | 2.6861535025 | 2.6542465756 | - |
| 0.40→0.45 | - | 2.8548668064 | 2.8143823001 | 2.7390546261 | 2.6733607264 | 2.6423773751 | - |
| 0.45→0.50 | - | 2.8330165333 | 2.7967124373 | 2.7243854531 | 2.6605788576 | 2.6305153199 | - |
| 0.50→0.55 | - | 2.8112131607 | 2.7790730827 | 2.7097348366 | 2.6478080378 | 2.6186605597 | - |
| 0.55→0.60 | - | 2.7894567861 | 2.7614643483 | 2.6951029055 | 2.6350484087 | 2.6068132433 | - |
| 0.60→0.65 | - | 2.7677475075 | 2.7438863459 | 2.6804897889 | 2.6223001113 | 2.5949735195 | - |
| ^*^ Unless otherwise indicated, $\alpha=$ 0.9, $\beta=$ 0.25, $\theta=$ 0.50, $K_{h}=$ 78.13, $K_{l}=$ 14.66, $\alpha_{a}=$ 0.9, $\kappa=$ 0.75, $R_{B}=$ 2.41%, $R_{g}=$ 4.60%, $R_{c}=$ 5.10%, $R_{h}=$ 5.60%, $R_{l}=$ 5.10%, $\sigma_{h}=$ 0.2904, $\sigma_{l}=$ 0.2404, $b_{h}=$ 0.03, $b_{l}=$ 0.03, $\delta=$ 0.85, $c_{h}=$ 0.038, $c_{cap}=$ 0.022, $c_{l}=$ 0.60%, $R_{ci}=$ 0.5%, $R_{a}=$ 5.6%, and $\sigma_{a}=$ 29.04%. The shaded areas illustrate the optimal values and the comparative statics evaluated at the optimal guaranteed rate. | | | | | | | |

| Table 4 | | | | | | | |
| --- | --- | --- | --- | --- | --- | --- | --- |
| Responsiveness of the optimal guaranteed rate to $c_{cap}$^*^$\beta$ $b_{h}$ | | | | | | | |
|  | $(R(\%), L)$ | | | | | | |
| $c_{cap}$ | (1.00, 307) | (1.20, 323) | (1.40, 334) | (1.60, 341) | (1.80, 345) | (2.00, 347) | (2.20, 348) |
|  | $S$ | | | | | | |
| 0.0145 | 33.1674933264 | 34.7897335257 | 35.8452041686 | 36.4590450262 | 36.7477964101 | 36.8225193206 | 36.7907078236 |
| 0.0170 | 33.1626065111 | 34.7886243534 | 35.8468965840 | 36.4626635545 | 36.7526446641 | 36.8281186193 | 36.7968155898 |
| 0.0195 | 33.1570809408 | 34.7868030924 | 35.8478273672 | 36.4654892512 | 36.7566822854 | 36.8328982754 | 36.8020990863 |
| 0.0220 | 33.1509267617 | 34.7842802017 | 35.8480071393 | 36.4675328085 | 36.7599199815 | 36.8368689795 | 36.8065689712 |
| 0.0245 | 33.1441540729 | 34.7810661047 | 35.8474464946 | 36.4688048972 | 36.7623684423 | 36.8400414073 | 36.8102358899 |
| 0.0270 | 33.1367729248 | 34.7771711871 | 35.8461559975 | 36.4693161642 | 36.7640383380 | 36.8424262171 | 36.8131104722 |
| 0.0295 | 33.1287933179 | 34.7726057950 | 35.8441461811 | 36.4690772307 | 36.7649403164 | 36.8440340473 | 36.8152033297 |
|  | $\partial^{2}S/\partial R^{2}$ | | | | | | |
| 0.0145 | - | -14.1692389100 | -11.0407446325 | -8.1272368425 | -5.3507118350 | -2.6633601875 | - |
| 0.0170 | - | -14.1936402925 | -11.0626315025 | -8.1446465225 | -5.3626788600 | -2.6694246175 | - |
| 0.0195 | - | -14.2174469200 | -11.0840597700 | -8.1617212450 | -5.3744261050 | -2.6753794775 | - |
| 0.0220 | - | -14.2406625600 | -11.1050317100 | -8.1784624050 | -5.3859543750 | -2.6812251575 | - |
| 0.0245 | - | -14.2632910475 | -11.1255496825 | -8.1948714375 | -5.3972645025 | -2.6869620600 | - |
| 0.0270 | - | -14.2853362975 | -11.1456160925 | -8.2109498225 | -5.4083573675 | -2.6925906000 | - |
| 0.0295 | - | -14.3068022750 | -11.1652334125 | -8.2266990975 | -5.4192338700 | -2.6981112125 | - |
|  | $\partial^{2}S/\partial R\partial c_{cap}$ | | | | | | |
| 0.0145→0.0170 | 7.5552860000 | 5.6031754000 | 3.8522258000 | 2.4594514000 | 1.5020894000 | 1.0169350000 | - |
| 0.0170→0.0195 | 7.4086186000 | 5.5040884000 | 3.7898270000 | 2.4238492000 | 1.4840696000 | 1.0076808000 | - |
| 0.0195→0.0220 | 7.2625768000 | 5.4053256000 | 3.7275704000 | 2.3882776000 | 1.4660160000 | 0.9983616000 | - |
| 0.0220→0.0245 | 7.1171836000 | 5.3069046000 | 3.6654668000 | 2.3527442000 | 1.4479340000 | 0.9889818000 | - |
| 0.0245→0.0270 | 6.9724610000 | 5.2088410000 | 3.6035282000 | 2.3172574000 | 1.4298282000 | 0.9795450000 | - |
| 0.0270→0.0295 | 6.8284296000 | 5.1111514000 | 3.5417658000 | 2.2818238000 | 1.4117036000 | 0.9700546000 | - |
|  | $\partial R/\partial c_{cap}$ | | | | | | |
| 0.0145→0.0170 | - | 0.5332174895 | 0.5074997735 | 0.4739896074 | 0.4596493842 | 0.5639828240 | - |
| 0.0170→0.0195 | - | 0.5219674761 | 0.4975387998 | 0.4653150986 | 0.4519847754 | 0.5559511178 | - |
| 0.0195→0.0220 | - | 0.5108214464 | 0.4876665872 | 0.4567137603 | 0.4443781631 | 0.5479656297 | - |
| 0.0220→0.0245 | - | 0.4997789653 | 0.4778828858 | 0.4481853212 | 0.4368295823 | 0.5400270082 | - |
| 0.0245→0.0270 | - | 0.4888395656 | 0.4681872940 | 0.4397296806 | 0.4293392327 | 0.5321356119 | - |
| 0.0270→0.0295 | - | 0.4780027196 | 0.4585795310 | 0.4313466623 | 0.4219069941 | 0.5242919588 | - |
| ^*^ Unless otherwise indicated, $\alpha=$ 0.9, $\beta=$ 0.25, $\theta=$ 0.50, $K_{h}=$ 78.13, $K_{l}=$ 14.66, $\alpha_{a}=$ 0.9, $\kappa=$ 0.75, $R_{B}=$ 2.41%, $R_{g}=$ 4.60%, $R_{c}=$ 5.10%, $R_{h}=$ 5.60%, $R_{l}=$ 5.10%, $\sigma_{h}=$ 0.2904, $\sigma_{l}=$ 0.2404, $b_{h}=$ 0.03, $b_{l}=$ 0.03, $\delta=$ 0.85, $c_{h}=$ 0.038, $c_{cap}=$ 0.022, $c_{l}=$ 0.60%, $R_{ci}=$ 0.5%, $R_{a}=$ 5.6%, and $\sigma_{a}=$ 29.04%. The shaded areas illustrate the optimal values and the comparative statics evaluated at the optimal guaranteed rate. | | | | | | | |

| Table 5 | | | | | | | |
| --- | --- | --- | --- | --- | --- | --- | --- |
| Responsiveness of $PUT_{lia}$ to $c_{cap}$^*^ | | | | | | | |
|  | $(R(\%), L)$ | | | | | | |
| $c_{cap}$ | (1.00, 307) | (1.20, 323) | (1.40, 334) | (1.60, 341) | (1.80, 345) | (2.00, 347) | (2.20, 348) |
|  | $PUT_{lia}$ | | | | | | |
| 0.0145 | 372.8642862626 | 390.6580897233 | 402.9078160716 | 410.7440460868 | 415.2783272600 | 417.6136466764 | 418.8501170685 |
| 0.0170 | 373.1715321886 | 390.9880642813 | 403.2532388208 | 411.0991626348 | 415.6388553456 | 417.9767445742 | 419.2143669667 |
| 0.0195 | 373.4767901861 | 391.3159756929 | 403.5965485659 | 411.4521351989 | 415.9972220758 | 418.3376726352 | 419.5764429331 |
| 0.0220 | 373.7800652819 | 391.6418285653 | 403.9377496533 | 411.8029679828 | 416.3534315951 | 418.6964349981 | 419.9363491271 |
| 0.0245 | 374.0813625939 | 391.9656276012 | 404.2768465270 | 412.1516652883 | 416.7074881459 | 419.0530358986 | 420.2940898042 |
| 0.0270 | 374.3806873298 | 392.2873775977 | 404.6138437274 | 412.4982315146 | 417.0593960676 | 419.4074796690 | 420.6496693156 |
| 0.0295 | 374.6780447864 | 392.6070834455 | 404.9487458909 | 412.8426711574 | 417.4091597962 | 419.7597707374 | 421.0030921072 |
|  | $\partial PUT_{lia}/\partial c_{cap}:$direct effect | | | | | | |
| 0.0145→0.0170 | 122.8983704000 | 131.9898232000 | 138.1690996800 | 142.0466192000 | 144.2112342400 | 145.2391591200 | 145.6999592800 |
| 0.0170→0.0195 | 122.1031990000 | 131.1645646400 | 137.3238980400 | 141.1890256400 | 143.3466920800 | 144.3712244000 | 144.8303865600 |
| 0.0195→0.0220 | 121.3100383200 | 130.3411489600 | 136.4804349600 | 140.3331135600 | 142.4838077200 | 143.5049451600 | 143.9624776000 |
| 0.0220→0.0245 | 120.5189248000 | 129.5196143600 | 135.6387494800 | 139.4789222000 | 141.6226203200 | 142.6403602000 | 143.0962708400 |
| 0.0245→0.0270 | 119.7298943600 | 128.6999986000 | 134.7988801600 | 138.6264905200 | 140.7631686800 | 141.7775081600 | 142.2318045600 |
| 0.0270→0.0295 | 118.9429826400 | 127.8823391200 | 133.9608654000 | 137.7758571200 | 139.9054914400 | 140.9164273600 | 141.3691166400 |
|  | $(\partial PUT_{lia}/\partial R)(\partial R/\partial c_{cap}):$indirect effect | | | | | | |
| 0.016→0.018 | - | 47.4398360516 | 31.0836667345 | 18.5714579409 | 10.4208977454 | 6.5854001975 | - |
| 0.018→0.020 | - | 46.4982514455 | 30.5120010965 | 18.2541340672 | 10.2593599510 | 6.4987606498 | - |
| 0.020→0.022 | - | 45.5631927172 | 29.9441253073 | 17.9387725510 | 10.0986867863 | 6.4124323225 | - |
| 0.022→0.024 | - | 44.6346678647 | 29.3800512628 | 17.6253770170 | 9.9388855956 | 6.3264255891 | - |
| 0.024→0.026 | - | 43.7126816882 | 28.8197813729 | 17.3139576946 | 9.7799674492 | 6.2407474433 | - |
| 0.026→0.028 | - | 42.7972332316 | 28.2633252811 | 17.0045217802 | 9.6219361310 | 6.1554067544 | - |
|  | $dPUT_{lia}/dc_{cap}:$total effect | | | | | | |
| 0.016→0.018 | - | 179.4296592516 | 169.2527664145 | 160.6180771409 | 154.6321319854 | 151.8245593175 | - |
| 0.018→0.020 | - | 177.6628160854 | 167.8358991365 | 159.4431597072 | 153.6060520310 | 150.8699850498 | - |
| 0.020→0.022 | - | 175.9043416772 | 166.4245602673 | 158.2718861110 | 152.5824945063 | 149.9173774825 | - |
| 0.022→0.024 | - | 174.1542822247 | 165.0188007428 | 157.1042992170 | 151.5615059156 | 148.9667857891 | - |
| 0.024→0.026 | - | 172.4126802882 | 163.6186615329 | 155.9404482145 | 150.5431361292 | 148.0182556033 | - |
| 0.026→0.028 | - | 170.6795723516 | 162.2241906811 | 154.7803789002 | 149.5274275710 | 147.0718341144 | - |
| ^*^ Unless otherwise indicated, $\alpha=$ 0.9, $\beta=$ 0.25, $\theta=$ 0.50, $K_{h}=$ 78.13, $K_{l}=$ 14.66, $\alpha_{a}=$ 0.9, $\kappa=$ 0.75, $R_{B}=$ 2.41%, $R_{g}=$ 4.60%, $R_{c}=$ 5.10%, $R_{h}=$ 5.60%, $R_{l}=$ 5.10%, $\sigma_{h}=$ 0.2904, $\sigma_{l}=$ 0.2404, $b_{h}=$ 0.03, $b_{l}=$ 0.03, $\delta=$ 0.85, $c_{h}=$ 0.038, $c_{cap}=$ 0.022, $c_{l}=$ 0.60%, $R_{ci}=$ 0.5%, $R_{a}=$ 5.6%, and $\sigma_{a}=$ 29.04%. The shaded areas illustrate the optimal values and the comparative statics evaluated at the optimal guaranteed rate. | | | | | | | |

| Table 6 | | | | | | | |
| --- | --- | --- | --- | --- | --- | --- | --- |
| Responsiveness of the optimal guaranteed rate to $\beta$^*^$b_{h}$ | | | | | | | |
|  | $(R(\%), L)$ | | | | | | |
| $\beta$ | (1.00, 307) | (1.20, 323) | (1.40, 334) | (1.60, 341) | (1.80, 345) | (2.00, 347) | (2.20, 348) |
|  | $S$ | | | | | | |
| 0.175 | 26.3549279709 | 27.6607129562 | 28.5013609857 | 28.9789241226 | 29.1891136384 | 29.2233363504 | 29.1699751929 |
| 0.200 | 28.6633897499 | 30.0819745712 | 30.9993998771 | 31.5257527673 | 31.7641036449 | 31.8128621307 | 31.7672481498 |
| 0.225 | 30.9460538840 | 32.4730517969 | 33.4641839555 | 34.0373685838 | 34.3027806408 | 34.3655542916 | 34.3274583120 |
| 0.250 | 33.1974719129 | 34.8279387732 | 35.8893324390 | 36.5071565676 | 36.7983981542 | 36.8746023005 | 36.8437645706 |
| 0.275 | 35.4117632754 | 37.1401897890 | 38.2680223836 | 38.9280592488 | 39.2437673937 | 39.3327533705 | 39.3088835094 |
| 0.300 | 37.5826463653 | 39.4029605858 | 40.5930373416 | 41.2926301141 | 41.6313133829 | 41.7323699013 | 41.7151474436 |
| 0.325 | 39.7034851523 | 41.6090670731 | 42.8568346317 | 43.5931063668 | 43.9531508236 | 44.0655068280 | 44.0545824401 |
|  | $\partial^{2}S/\partial R^{2}$ | | | | | | |
| 0.175 | - | -11.6284238936 | -9.0771223154 | -6.6843405269 | -4.3991700960 | -2.1895967369 | - |
| 0.200 | - | -12.5289878872 | -9.7768103923 | -7.2000503146 | -4.7398097934 | -2.3593116692 | - |
| 0.225 | - | -13.3966438529 | -10.4486882616 | -7.6943142789 | -5.0659601573 | -2.5217407610 | - |
| 0.250 | - | -14.2268298631 | -11.0892384301 | -8.1645635501 | -5.3759360076 | -2.6760469051 | - |
| 0.275 | - | -15.0148479776 | -11.6948932324 | -8.6082180082 | -5.6680542029 | -2.8213959467 | - |
| 0.300 | - | -15.7559366164 | -12.2620995829 | -9.0227375928 | -5.9406687590 | -2.9569744042 | - |
| 0.325 | - | -16.4453590520 | -12.7873955897 | -9.4056819586 | -6.1922113085 | -3.0820098059 | - |
|  | $\partial^{2}S/\partial R\partial\beta$ | | | | | | |
| 0.175→0.200 | 22.5599672189 | 15.3554552705 | 9.7579506550 | 5.6322723532 | 2.9071547737 | 1.5494353155 | - |
| 0.200→0.225 | 21.6826182903 | 14.7413705644 | 9.3663476096 | 5.4122358956 | 2.8030329844 | 1.5036002494 | - |
| 0.225→0.250 | 20.6937895057 | 14.0523014241 | 8.9279000761 | 5.1659059063 | 2.6860991041 | 1.4516499512 | - |
| 0.250→0.275 | 19.5919306617 | 13.2877857457 | 8.4425473273 | 4.8933116626 | 2.5563661000 | 1.3935737676 | - |
| 0.275→0.300 | 18.3775413663 | 12.4488322560 | 7.9111814522 | 4.5950247754 | 2.4141083264 | 1.3294806660 | - |
| 0.300→0.325 | 17.0535400556 | 11.5381605711 | 7.3357925170 | 4.2722375900 | 2.2598971944 | 1.2596139809 | - |
|  | $\partial R/\partial\beta$ | | | | | | |
| 0.175→0.200 | - | 1.9400709353 | 1.6916655672 | 1.4598224934 | 1.2803033823 | 1.3277124160 | - |
| 0.200→0.225 | - | 1.7305961571 | 1.5077893477 | 1.3008725218 | 1.1418677397 | 1.1880723607 | - |
| 0.225→0.250 | - | 1.5446995332 | 1.3448866568 | 1.1603243320 | 1.0197288857 | 1.0651765422 | - |
| 0.250→0.275 | - | 1.3771114753 | 1.1982595405 | 1.0340475980 | 0.9102250577 | 0.9552770152 | - |
| 0.275→0.300 | - | 1.2239578711 | 1.0644673712 | 0.9190266144 | 0.8106882205 | 0.8556432248 | - |
| 0.300→0.325 | - | 1.0823564775 | 0.9409612516 | 0.8130340090 | 0.7191509514 | 0.7642599784 | - |
| ^*^ Unless otherwise indicated, $\alpha=$ 0.9, $\beta=$ 0.25, $\theta=$ 0.50, $K_{h}=$ 78.13, $K_{l}=$ 14.66, $\alpha_{a}=$ 0.9, $\kappa=$ 0.75, $R_{B}=$ 2.41%, $R_{g}=$ 4.60%, $R_{c}=$ 5.10%, $R_{h}=$ 5.60%, $R_{l}=$ 5.10%, $\sigma_{h}=$ 0.2904, $\sigma_{l}=$ 0.2404, $b_{h}=$ 0.03, $b_{l}=$ 0.03, $\delta=$ 0.85, $c_{h}=$ 0.038, $c_{cap}=$ 0.022, $c_{l}=$ 0.60%, $R_{ci}=$ 0.5%, $R_{a}=$ 5.6%, and $\sigma_{a}=$ 29.04%. The shaded areas illustrate the optimal values and the comparative statics evaluated at the optimal guaranteed rate. | | | | | | | |

| Table 7 | | | | | | | |
| --- | --- | --- | --- | --- | --- | --- | --- |
| Responsiveness of $PUT_{lia}$ to $\beta$^*^ | | | | | | | |
|  | $(R(\%), L)$ | | | | | | |
| $\beta$ | (1.00, 307) | (1.20, 323) | (1.40, 334) | (1.60, 341) | (1.80, 345) | (2.00, 347) | (2.20, 348) |
|  | $PUT_{lia}$ | | | | | | |
| 0.175 | 361.1515810720 | 378.6674310930 | 390.7296496870 | 398.4485217790 | 402.9172068952 | 405.2211505307 | 406.4432821942 |
| 0.200 | 365.4134183465 | 383.0455314949 | 395.1862507610 | 402.9543055193 | 407.4505389867 | 409.7677297184 | 410.9959880177 |
| 0.225 | 369.5258100457 | 387.2626837058 | 399.4740573343 | 407.2863125532 | 411.8072317758 | 414.1362097560 | 415.3698338837 |
| 0.250 | 373.4837018019 | 391.3138731087 | 403.5881004639 | 411.4396093961 | 415.9823732990 | 418.3216887388 | 419.5599217026 |
| 0.275 | 377.2825205177 | 395.1946603974 | 407.5240519359 | 415.4099451968 | 419.9717583213 | 422.3199836685 | 423.5620784962 |
| 0.300 | 380.9182753377 | 398.9012909445 | 411.2783390479 | 419.1938698409 | 423.7720083287 | 426.1277514344 | 427.3729779269 |
| 0.325 | 384.3876615967 | 402.4308060572 | 414.8482604505 | 422.7888525598 | 427.3806917138 | 429.7426098425 | 430.9902618163 |
|  | $\partial PUT_{lia}/\partial\beta:$direct effect | | | | | | |
| 0.175→0.200 | 170.4734909788 | 175.1240160793 | 178.2640429583 | 180.2313496124 | 181.3332836626 | 181.8631675068 | 182.1082329393 |
| 0.200→0.225 | 164.4956679706 | 168.6860884366 | 171.5122629345 | 173.2802813593 | 174.2677115610 | 174.7392015065 | 174.9538346397 |
| 0.225→0.250 | 158.3156702449 | 162.0475761138 | 164.5617251838 | 166.1318737149 | 167.0056609273 | 167.4191593118 | 167.6035127562 |
| 0.250→0.275 | 151.9527486322 | 155.2314915493 | 157.4380588782 | 158.8134320286 | 159.5754008922 | 159.9317971852 | 160.0862717439 |
| 0.275→0.300 | 145.4301927998 | 148.2652218839 | 150.1714844811 | 151.3569857612 | 152.0100002973 | 152.3107106361 | 152.4359772299 |
| 0.300→0.325 | 138.7754503623 | 141.1806045087 | 142.7968561054 | 143.7993087591 | 144.3473354047 | 144.5943363245 | 144.6913555742 |
|  | $(\partial PUT_{lia}/\partial R)(\partial R/\partial\beta):$indirect effect | | | | | | |
| 0.175→0.200 | - | 169.9099576600 | 102.0261993000 | 56.3409155194 | 28.6063633438 | 15.2948728537 | - |
| 0.200→0.225 | - | 152.5703362836 | 91.5282359162 | 50.5262449132 | 25.6705197341 | 13.7649513136 | - |
| 0.225→0.250 | - | 136.9907023137 | 82.1145672701 | 45.3237490928 | 23.0505596060 | 12.4038635593 | - |
| 0.250→0.275 | - | 122.7706675664 | 73.5385501527 | 40.5941697582 | 20.6746876777 | 11.1734713549 | - |
| 0.275→0.300 | - | 109.6185229743 | 65.6211749993 | 36.2367289263 | 18.4910408201 | 10.0462155436 | - |
| 0.300→0.325 | - | 97.3201671336 | 58.2316133739 | 32.1779786690 | 16.4618632459 | 9.0020008756 | - |
|  | $dPUT_{lia}/d\beta:$total effect | | | | | | |
| 0.175→0.200 | - | 345.0339737393 | 280.2902422583 | 236.5722651317 | 209.9396470064 | 197.1580403605 | - |
| 0.200→0.225 | - | 321.2564247201 | 263.0404988507 | 223.8065262725 | 199.9382312951 | 188.5041528201 | - |
| 0.225→0.250 | - | 299.0382784275 | 246.6762924539 | 211.4556228078 | 190.0562205333 | 179.8230228711 | - |
| 0.250→0.275 | - | 278.0021591157 | 230.9766090309 | 199.4076017868 | 180.2500885699 | 171.1052685400 | - |
| 0.275→0.300 | - | 257.8837448582 | 215.7926594804 | 187.5937146874 | 170.5010411174 | 162.3569261797 | - |
| 0.300→0.325 | - | 238.5007716423 | 201.0284694793 | 175.9772874281 | 160.8091986506 | 153.5963372001 | - |
| ^*^ Unless otherwise indicated, $\alpha=$ 0.9, $\beta=$ 0.25, $\theta=$ 0.50, $K_{h}=$ 78.13, $K_{l}=$ 14.66, $\alpha_{a}=$ 0.9, $\kappa=$ 0.75, $R_{B}=$ 2.41%, $R_{g}=$ 4.60%, $R_{c}=$ 5.10%, $R_{h}=$ 5.60%, $R_{l}=$ 5.10%, $\sigma_{h}=$ 0.2904, $\sigma_{l}=$ 0.2404, $b_{h}=$ 0.03, $b_{l}=$ 0.03, $\delta=$ 0.85, $c_{h}=$ 0.038, $c_{cap}=$ 0.022, $c_{l}=$ 0.60%, $R_{ci}=$ 0.5%, $R_{a}=$ 5.6%, and $\sigma_{a}=$ 29.04%. The shaded areas illustrate the optimal values and the comparative statics evaluated at the optimal guaranteed rate. | | | | | | | |

| Table 8 | | | | | | | |
| --- | --- | --- | --- | --- | --- | --- | --- |
| Responsiveness of the optimal guaranteed rate to $b_{h}$^*^ | | | | | | | |
|  | $(R(\%), L)$ | | | | | | |
| $b_{h}$ | (1.00, 307) | (1.20, 323) | (1.40, 334) | (1.60, 341) | (1.80, 345) | (2.00, 347) | (2.20, 348) |
|  | $S$ | | | | | | |
| 0.015 | 33.4511983618 | 35.1118367333 | 36.1936619671 | 36.8243881384 | 37.1230241079 | 37.2030056901 | 37.1741418981 |
| 0.020 | 33.3670317537 | 35.0175008369 | 36.0924330111 | 36.7188008887 | 37.0149335519 | 37.0936303544 | 37.0640894041 |
| 0.025 | 33.2824493739 | 34.9228613847 | 35.9909831599 | 36.6130504679 | 36.9067184251 | 36.9841561504 | 36.9539572785 |
| 0.030 | 33.1974719129 | 34.8279387732 | 35.8893324390 | 36.5071565676 | 36.7983981542 | 36.8746023005 | 36.8437645706 |
| 0.035 | 33.1121198103 | 34.7327530926 | 35.7875005297 | 36.4011385099 | 36.6899917792 | 36.7649876284 | 36.7335299207 |
| 0.040 | 33.0264132422 | 34.6373241158 | 35.6855067608 | 36.2950152404 | 36.5815179473 | 36.6553305531 | 36.6232715553 |
| 0.045 | 32.9403721090 | 34.5416712888 | 35.5833701000 | 36.1888053214 | 36.4729949072 | 36.5456490843 | 36.5130072829 |
|  | $\partial^{2}S/\partial R^{2}$ | | | | | | |
| 0.015 | - | -14.4703284410 | -11.2774765603 | -8.3022550485 | -5.4663596810 | -2.7211343549 | - |
| 0.020 | - | -14.3884227257 | -11.2141074136 | -8.2558803598 | -5.4358965181 | -2.7059438200 | - |
| 0.025 | - | -14.3072558871 | -11.1513616813 | -8.2099837701 | -5.4057557996 | -2.6909149268 | - |
| 0.030 | - | -14.2268298631 | -11.0892384301 | -8.1645635501 | -5.3759360076 | -2.6760469051 | - |
| 0.035 | - | -14.1471461309 | -11.0277364235 | -8.1196177724 | -5.3464355022 | -2.6613389235 | - |
| 0.040 | - | -14.0682057184 | -10.9668541318 | -8.0751443194 | -5.3172525273 | -2.6467900918 | - |
| 0.045 | - | -13.9900092170 | -10.9065897436 | -8.0311408919 | -5.2883852161 | -2.6323994643 | - |
|  | $\partial^{2}S/\partial R\partial b_{h}$ | | | | | | |
| 0.015→0.020 | -10.1692882531 | -6.8930596393 | -4.3582937705 | -2.5033062227 | -1.2847797055 | -0.6771583107 | - |
| 0.020→0.025 | -10.0570724394 | -6.8103988973 | -4.3005696059 | -2.4647060158 | -1.2590772755 | -0.6579215456 | - |
| 0.025→0.030 | -9.9451504097 | -6.7281094498 | -4.2431794026 | -2.4263706033 | -1.2335789236 | -0.6388580577 | - |
| 0.030→0.035 | -9.8335779752 | -6.6462286846 | -4.1861484172 | -2.3883173078 | -1.2082970931 | -0.6199778289 | - |
| 0.035→0.040 | -9.7224086853 | -6.5647921882 | -4.1295005229 | -2.3505624030 | -1.1832434064 | -0.6012901387 | - |
| 0.040→0.045 | -9.6116938172 | -6.4838337586 | -4.0732582315 | -2.3131211348 | -1.1584286866 | -0.5828035840 | - |
|  | $\partial R/\partial b_{h}$ | | | | | | |
| 0.015→0.020 | - | -0.7027683093 | -0.6112235838 | -0.5249530092 | -0.4579475865 | -0.4721485741 | - |
| 0.020→0.025 | - | -0.6989697642 | -0.6073063728 | -0.5209098750 | -0.4534129757 | -0.4653005972 | - |
| 0.025→0.030 | - | -0.6951123603 | -0.6033442051 | -0.5168316432 | -0.4488494659 | -0.4584236058 | - |
| 0.030→0.035 | - | -0.6911995202 | -0.5993404080 | -0.5127216405 | -0.4442607398 | -0.4515231369 | - |
| 0.035→0.040 | - | -0.6872346264 | -0.5952982494 | -0.5085831179 | -0.4396503805 | -0.4446045545 | - |
| 0.040→0.045 | - | -0.6832210169 | -0.5912209354 | -0.5044192488 | -0.4350218695 | -0.4376730479 | - |
| ^*^ Unless otherwise indicated, $\alpha=$ 0.9, $\beta=$ 0.25, $\theta=$ 0.50, $K_{h}=$ 78.13, $K_{l}=$ 14.66, $\alpha_{a}=$ 0.9, $\kappa=$ 0.75, $R_{B}=$ 2.41%, $R_{g}=$ 4.60%, $R_{c}=$ 5.10%, $R_{h}=$ 5.60%, $R_{l}=$ 5.10%, $\sigma_{h}=$ 0.2904, $\sigma_{l}=$ 0.2404, $b_{h}=$ 0.03, $b_{l}=$ 0.03, $\delta=$ 0.85, $c_{h}=$ 0.038, $c_{cap}=$ 0.022, $c_{l}=$ 0.60%, $R_{ci}=$ 0.5%, $R_{a}=$ 5.6%, and $\sigma_{a}=$ 29.04%. The shaded areas illustrate the optimal values and the comparative statics evaluated at the optimal guaranteed rate. | | | | | | | |

| Table 9 | | | | | | | |
| --- | --- | --- | --- | --- | --- | --- | --- |
| Responsiveness of $PUT_{lia}$ to $b_{h}$^*^ | | | | | | | |
|  | $(R(\%), L)$ | | | | | | |
| $b_{h}$ | (1.00, 307) | (1.20, 323) | (1.40, 334) | (1.60, 341) | (1.80, 345) | (2.00, 347) | (2.20, 348) |
|  | $PUT_{lia}$ | | | | | | |
| 0.015 | 374.2786218779 | 392.1387570366 | 404.4327803370 | 412.2965457775 | 416.8461446943 | 419.1887584207 | 420.4285457080 |
| 0.020 | 374.0151251864 | 391.8654187948 | 404.1529535910 | 412.0127103620 | 416.5600809969 | 418.9016268511 | 420.1409182078 |
| 0.025 | 373.7501573142 | 391.5904630582 | 403.8713991930 | 411.7270704133 | 416.2721626556 | 418.6126090291 | 419.8513822281 |
| 0.030 | 373.4837018019 | 391.3138731087 | 403.5881004639 | 411.4396093961 | 415.9823732990 | 418.3216887388 | 419.5599217026 |
| 0.035 | 373.2157424173 | 391.0356325216 | 403.3030410625 | 411.1503111424 | 415.6906969424 | 418.0288501649 | 419.2665209758 |
| 0.040 | 372.9462631695 | 390.7557251774 | 403.0162049948 | 410.8591598597 | 415.3971179951 | 417.7340778984 | 418.9711648090 |
| 0.045 | 372.6752483214 | 390.4741352723 | 402.7275766234 | 410.5661401381 | 415.1016212667 | 417.4373569428 | 418.6738383845 |
|  | $\partial PUT_{lia}/\partial b_{h}:$direct effect | | | | | | |
| 0.015→0.020 | -52.6993383045 | -54.6676483627 | -55.9653492038 | -56.7670830880 | -57.2127394914 | -57.4263139271 | -57.5255000295 |
| 0.020→0.025 | -52.9935744414 | -54.9911473185 | -56.3108795989 | -57.1279897396 | -57.5836682592 | -57.8035644025 | -57.9071959389 |
| 0.025→0.030 | -53.2911024589 | -55.3179899076 | -56.6597458169 | -57.4922034390 | -57.9578713262 | -58.1840580523 | -58.2921051090 |
| 0.030→0.035 | -53.5918769089 | -55.6481174187 | -57.0118802903 | -57.8596507360 | -58.3352713213 | -58.5677147941 | -58.6801453511 |
| 0.035→0.040 | -53.8958495626 | -55.9814688336 | -57.3672135231 | -58.2302565401 | -58.7157894498 | -58.9544532894 | -59.0712333625 |
| 0.040→0.045 | -54.2029696130 | -56.3179810324 | -57.7256742927 | -58.6039443181 | -59.0993456861 | -59.3441911316 | -59.4652849108 |
|  | $(\partial PUT_{lia}/\partial R)(\partial R/\partial b_{h}):$indirect effect | | | | | | |
| 0.015→0.020 | - | -62.7576849441 | -37.5719849039 | -20.6405366563 | -10.4173892170 | -5.5303086527 | - |
| 0.020→0.025 | - | -62.3840775693 | -37.3114909374 | -20.4711245842 | -10.3091842558 | -5.4476134219 | - |
| 0.025→0.030 | - | -62.0050851749 | -37.0481582527 | -20.3002973266 | -10.2003111264 | -5.3645793284 | - |
| 0.030→0.035 | - | -61.6210292608 | -36.7822021519 | -20.1281926997 | -10.0908582608 | -5.2812752279 | - |
| 0.035→0.040 | - | -61.2322275878 | -36.5138341477 | -19.9549454216 | -9.9809117231 | -5.1977678589 | - |
| 0.040→0.045 | - | -60.8389937194 | -36.2432617326 | -19.7806870065 | -9.8705551579 | -5.1141218181 | - |
|  | $dPUT_{lia}/db_{h}:$total effect | | | | | | |
| 0.015→0.020 | - | -117.4253333068 | -93.5373341078 | -77.4076197444 | -67.6301287084 | -62.9566225797 | - |
| 0.020→0.025 | - | -117.3752248878 | -93.6223705363 | -77.5991143238 | -67.8928525150 | -63.2511778245 | - |
| 0.025→0.030 | - | -117.3230750825 | -93.7079040696 | -77.7925007656 | -68.1581824526 | -63.5486373807 | - |
| 0.030→0.035 | - | -117.2691466794 | -93.7940824422 | -77.9878434357 | -68.4261295821 | -63.8489900221 | - |
| 0.035→0.040 | - | -117.2136964214 | -93.8810476708 | -78.1852019617 | -68.6967011729 | -64.1522211482 | - |
| 0.040→0.045 | - | -117.1569747518 | -93.9689360253 | -78.3846313246 | -68.9699008440 | -64.4583129497 | - |
| ^*^ Unless otherwise indicated, $\alpha=$ 0.9, $\beta=$ 0.25, $\theta=$ 0.50, $K_{h}=$ 78.13, $K_{l}=$ 14.66, $\alpha_{a}=$ 0.9, $\kappa=$ 0.75, $R_{B}=$ 2.41%, $R_{g}=$ 4.60%, $R_{c}=$ 5.10%, $R_{h}=$ 5.60%, $R_{l}=$ 5.10%, $\sigma_{h}=$ 0.2904, $\sigma_{l}=$ 0.2404, $b_{h}=$ 0.03, $b_{l}=$ 0.03, $\delta=$ 0.85, $c_{h}=$ 0.038, $c_{cap}=$ 0.022, $c_{l}=$ 0.60%, $R_{ci}=$ 0.5%, $R_{a}=$ 5.6%, and $\sigma_{a}=$ 29.04%. The shaded areas illustrate the optimal values and the comparative statics evaluated at the optimal guaranteed rate. | | | | | | | |
